# CD63-positive extracellular vesicles are potential diagnostic biomarkers of pancreatic ductal adenocarcinoma

**DOI:** 10.1186/s12876-022-02228-7

**Published:** 2022-03-28

**Authors:** Haruki Odaka, Keiko Hiemori, Asako Shimoda, Kazunari Akiyoshi, Hiroaki Tateno

**Affiliations:** 1 Cellular and Molecular Biotechnology Research Institute, National Institute of Advanced Industrial Science and Technology, 1-1-1 Higashi, Tsukuba, Ibaraki 305-8566 Japan; 2 Department of Polymer Chemistry, Graduate School of Engineering, Kyoto University, Katsura, Nishikyo-ku, Kyoto, 615-8510 Japan

**Keywords:** Pancreatic ductal adenocarcinoma, Extracellular vesicle, CD63, Platelet, Exosome, Biomarker

## Abstract

**Background:**

Pancreatic ductal adenocarcinoma (PDAC) is one of the deadliest gastrointestinal cancers with a 5-year survival rate of less than 10%. Biomarkers for early PDAC detection are useful in treating patients with PDAC. Extracellular vesicles (EVs) are lipid-bound vesicles that are potential biomarkers of various diseases such as PDAC. In this study, we quantitatively measured the serum levels of EVs (CD63^+^-EVs) or platelet-derived EVs (CD41^+^- and CD61^+^-EVs) and evaluated their potential use as biomarkers of PDAC.

**Methods:**

We measured the serum levels of CD63^+^-, CD41^+^-, CD61^+^-EVs using sandwich enzyme-linked immunosorbent assay based on Tim4 with specificity for phosphatidylserine on EVs in age- and sex-matched healthy controls (HCs, n = 39) and patients with PDAC (n = 39). We also examined the effect of tumor burden on the serum EV levels after surgical resection (n = 28). CA19-9, a clinical PDAC biomarker, was also measured for comparison.

**Results:**

Serum levels of CD63^+^-EVs, CD41^+^-EVs, and CD61^+^-EVs were significantly increased in patients with PDAC compared to HCs. Receiver operating characteristic analysis revealed that CD63^+^-EVs exhibited the highest diagnostic performance to discriminate patients with PDAC from HCs (area under the curve (AUC): 0.846), which was comparable to CA19-9 (AUC: 0.842). CA19-9 showed lower AUC values in early stages (I–II, AUC: 0.814) than in late stages (III–IV, AUC: 0.883) PDAC. Conversely, CD63^+^-EVs, CD41^+^-EVs, and CD61^+^-EVs showed comparable AUCs between early- and late-stage PDAC. The combined use of CA19-9 and CD63^+^-EVs showed a higher diagnostic performance for early-stage PDAC (AUC: 0.903) than CA19-9. The serum levels of CD63^+^-EVs, CD41^+^-EVs, CD61^+^-EVs, and CA19-9 decreased significantly after surgical resection, demonstrating that EVs are increased in sera of patients depending on the tumor burden.

**Conclusions:**

The serum levels of CD63^+^-EVs and platelet-derived EVs (CD41^+^-EVs, CD61^+^-EVs) are increased in patients with PDAC than HCs. Since CD63^+^-EVs showed a high AUC to discriminate patients with PDAC from HCs; they might be useful as potential biomarkers for PDAC.

**Supplementary Information:**

The online version contains supplementary material available at 10.1186/s12876-022-02228-7.

## Introduction

Extracellular vesicles (EVs) are nano- to micrometer-sized vesicles surrounded by lipid bilayers, and released from all cells. EVs are currently categorized into three groups: exosomes of endocytic origin, microvesicles generated by plasma membrane budding, and apoptotic bodies [1], although there is no unique marker for these EVs. Even the same cell was reported to release EVs with different compositions and functions [2]. EVs act as important mediators of intercellular communication in normal physiology and pathology [3]. EVs are also expected to be used for cancer detection, prognosis, and therapy [4].

Pancreatic ductal adenocarcinoma (PDAC) is one of the most lethal malignancies and has a dismal prognosis with an overall 5-year survival rate of less than 10% [3]. The only curative therapy for PDAC is early detection followed by surgical resection [5]. Hence, early detection is important in improving the prognosis and overall survival of patients with PDAC. EVs are potential diagnostic markers of PDAC. Previously, it was reported that glypican-1 (GPC1), a heparan sulfate proteoglycan, was specifically enriched on PDAC cell-derived EVs. GPC1^+^-EVs showed a high diagnostic performance to even early-stage PDAC [6]. miRNAs enriched with PDAC-derived EVs are intended to be used as important tools to diagnose PDAC [7, 8]. In addition, PDAC-derived EVs have been reported to promote proliferation, invasion, and metastasis of PDAC cells [7].

Previously we performed glycan profiling of EVs in sera of patients with Alzheimer’s disease (AD). EVs expressing CD61, a platelet marker, were only detected in sera of patients with AD, but not in healthy controls (HCs) [9]. Subsequent quantitative analysis using Tim4　(T Cell Immunoglobulin and Mucin Domain-containing Protein 4)-based sandwich enzyme-linked immunosorbent assay (ELISA) revealed that platelet marker (CD41, CD61)-positive EVs as well as tetraspanin (CD63 and CD9)-positive EVs are significantly increased in sera of patients with AD than those of HCs. Furthermore, among the EVs, CD63^+^-EVs showed the highest value for the area under the curve (AUC: 0.957) for discriminating patients with AD from HCs. However, we hypothesized that the upregulation of CD63^+^-EVs and platelet markers (CD41 and CD61)-positive EVs might not be an AD-specific pathology but common in various diseases such as cancers.

This study measured the serum levels of platelet-derived EVs (CD41^+^-, CD61^+^-EVs) and CD63^+^-EVs in sera of patients with PDAC using Tim4-based sandwich ELISA. We clearly demonstrate for the first time that all of these EVs are increased in sera of patients with PDAC than HCs. The serum levels of these EVs decreased after surgical resection, suggesting that the upregulation of the serum EVs depends on tumor burden. Among the EVs, CD63^+^-EVs provided the highest AUC (0.846) to discriminate patients with PDAC from HCs. The combined use of CA19-9 and CD63^+^-EVs showed a higher diagnostic performance for early-stage PDAC (AUC: 0.903) than CA19-9. Hence, CD63^+^-EVs might be used as a potential biomarker for PDAC.

## Methods

### Study population

This study involves two independent cohorts. The first cohort consists of 39 HCs and 39 patients with PDAC whose sera were obtained from BioIVT (Westbury, NY, USA) or Tsukuba University Hospital. The second cohort consists of 28 patients with PDAC whose sera were collected at presurgical operation and 30–90 days postoperation at Tsukuba University Hospital. All clinical samples were collected following the written informed consent. This study was approved by the medical ethics committee of Tsukuba University Hospital (H28-090) and the Committee for the Ethics on the Experiments with Human Derivative Samples of National Institute of Advanced Industrial Science and Technology. All experiments were performed in accordance with the ethical standards laid down in the 1964 Declaration of Helsinki and its later amendments. The demographic characteristics of participants are shown in Table 1 and Additional file 2: Table S1.

**Table 1:** Participant's characteristics of demographic data

	Cohort 1			Cohort 2
	HC	PDAC	*p-value*^a^	PDAC
N	39	39		28
Sex (male/female)	33/6	34/5	1	15/13
Age (year)	59.05 ± 8.772^b^	63.58 ± 10.83^b^	0.06065	70.07 ± 7.850^b^
*Clinical stage (N)*				
0		0		2
I		5		4
II		18		21
III		4		0
IV		12		1

HC: Healthy control, PDAC: Pancreatic ductal adenocarcinoma

^a^*p*-value in Chi-squared test (sex) or Wilcoxon–Mann–Whitney test (age)

^b^Mean ± SD

### Characterization of Tim4-captured EVs

EVs were prepared from serum using MagCaptureTM Exosome Isolation Kit PS (Wako Pure Chemical Industries, Ltd., Osaka, Japan) following the manufacturer’s instructions. The size distribution of the prepared EVs were analyzed by nanoparticle tracking analysis (NTA). EVs were diluted to a concentration of 4–8 × 10^8^ particles per mL with PBS and analyzed in triplicates using a Nanosight LM10 system (Marvern Instruments Ltd., Worcestershire, UK) equipped with a blue laser. Morphology of EVs was examined using an HT7700-transmission electron microscopy (TEM). EVs (5 μL) were mixed with 4% paraformaldehyde (5 μL), incubated for 10 min on Formvar film for TEM (PVF-C10 STEM; Okenshoji Co., Ltd., Tokyo, Japan), and negatively stained with 2% phosphotungstic acid for 20 s. After drying, the samples were observed by TEM.

### Sandwich ELISA

Serum levels of EVs expressing either platelet markers (CD41, CD61) or EV marker (CD63) were quantified using the PS Capture Exosome ELISA Kit (Streptavidin HRP) (Wako Pure Chemical Industries, Ltd) as described previously [9]. Briefly, 100-fold diluted sera (100 μl) were incubated with Tim4-coated plates for 2 h at room temperature (RT). After washing, a biotinylated antibody for each marker protein was incubated for 1 h at RT. Then, horseradish peroxidase (HRP)-labeled streptavidin was incubated for 2 h at RT followed by washing. After incubation with 3,3’,5,5’-tetramethylbenzidine solutions for 30 min, a stop solution was applied. An optical density (OD) was measured at 450 nm as the dominant wavelength and 620 nm as the secondary wavelength. The following antibodies were biotinylated and used for sandwich assays: anti-CD41 mAb (1 μg/mL, clone No. 745201; R&D Systems), anti-CD61 mAb (1 μg/mL, clone No. 256809; R&D Systems), anti-CD63 (component of PS Capture Exosome ELISA Kit; Wako).

CA19-9 was measured using Accuraseed CA19-9 reagents (FUJIFILM Wako Pure Chemical Co., Osaka, Japan).

### Statistical analysis

Wilcoxon–Mann–Whitney test, Wilcoxon signed rank test, Chi-square test, Pearson’s product-moment correlation, Spearman’s rank correlation, DeLong’s test, and receiving operator curve (ROC) analysis were calculated using EZR on R commander version 1.41 [10]. Statistical significance was set at *p*-value < 0.05. The summary of power calculation when the alpha was set at 0.05 in each cohort was shown in Additional file 3: Table S2.

## Results

### CD63 as well as platelet marker (CD41, CD61)-positive EVs are increased in sera of patients with PDAC

**Fig. 1:**
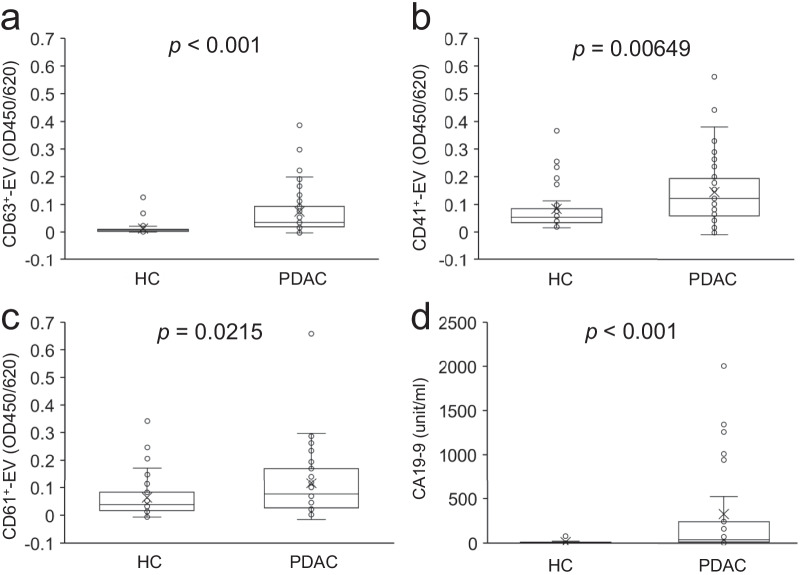
EVs are increased in sera of patients with PDAC relative to HCs. The serum levels of universal EVs [CD63 (**a**) and platelet-derived EVs (CD41(**b**), CD61(**c**)] in HC (n = 39) and patients with PDAC (n = 39) were evaluated with Tim4-based sandwich ELISA. CA19-9 level was also analyzed (**d**). OD: optical density. *p*-values obtained by Wilcoxon–Mann–Whitney test are indicated in the figure

We developed a sandwich ELISA based on Tim4 with affinity to phosphatidylserine on EVs to measure the serum levels of CD63 and platelet marker (CD41, CD61)-positive EVs. EVs were captured by Tim4 immobilized on 96 well plate and detected with biotinylated marker antibodies followed by HRP-conjugated streptavidin. Morphology and size distribution of Tim4-captured EVs isolated from sera of PDAC were examined by transmission electron microscope and nanoparticle tracking analysis, respectively (Additional file 1: Fig. S1a, b). Average diameters of Tim4-captured EVs was 170 ± 11.9 nm (average ± SD). We analyzed cohort 1 with HCs (n = 39) and patients with PDAC (n = 39), who showed no significant difference in age (*p* = 0.06065) and sex (*p* = 1) between them (Table 1). CD63^+^-EVs showed significantly higher serum levels in patients with PDAC than HCs (*p* < 0.001) (Fig. 1a). The serum levels of CD41^+^-EVs and CD61^+^-EVs were moderately but significantly increased in patients with PDAC relative to HCs (*p* = 0.00649 and 0.0215, respectively) (Fig. 1b, c). We also confirmed that the serum level of CA19-9, a clinically used biomarker of PDAC, is significantly increased in patients with PDAC than HCs (*p* < 0.001) (Fig. 1d). These results demonstrate that CD63 and platelet markers (CD41, CD61)-positive EVs are increased in patients with PDAC.

### Receiver operating characteristic (ROC) analysis

**Fig. 2:**
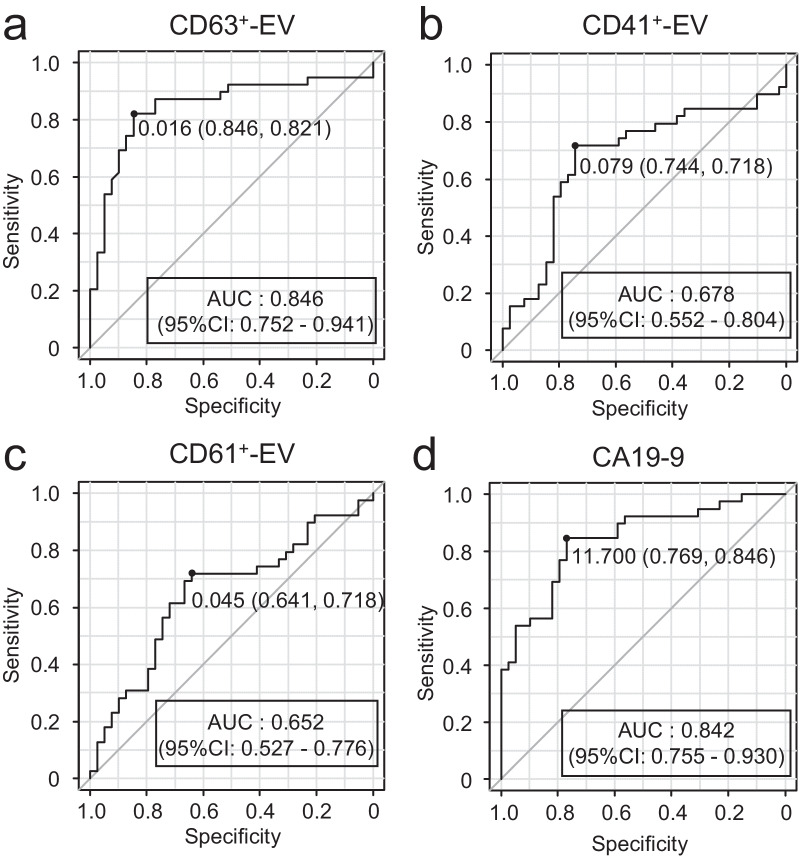
ROC curve of EVs and CA19-9. ROC curves are shown for CD63^+^-EVs (**a**), CD41^+^-EVs (**b**), CD61^+^-EVs (**c**), and CA19-9 (**d**). Area under curve (AUC) and its 95% confidence interval are indicated in the figure. Dot indicates the cut-off value which maximizes the sum of sensitivity and specificity

To evaluate the diagnostic ability of each marker-positive EV, we generated an ROC curve and quantified the AUC. Among the three types of EVs, CD63^+^-EVs showed the highest AUC value (0.846) and could discriminate patients with PDAC from HCs with high sensitivity (0.821) and specificity (0.846) when the cut-off value was set at 0.016 (Fig. 2a, Table 2). The AUC values of CD41^+^-EVs and CD61^+^-EVs were 0.678 and 0.652, respectively, showing moderate performance as diagnostic markers (Fig. 2b, c, Table 2). The AUC value of CA19-9 (0.842) was similar to that of CD63^+^-EVs (*p* = 0.945, Fig. 2d, Table 2), suggesting that CD63^+^-EVs show a similar diagnostic performance to discriminate PDAC from HCs.

**Table 2:** Summary of ROC analysis

	AUC (95% CI)	Threshold	Specificity	Sensitivity	*p*-value^a^ (vs CA19-9)
*All stage*					
CA19-9	0.842 (0.755–0.930)	11.7	0.769	0.846	
CD63^+^-EVs	0.846 (0.752–0.941)	0.016	0.846	0.821	0.945
CD41^+^-EVs	0.678 (0.552–0.804)	0.079	0.744	0.718	0.0290
CD61^+^-EVs	0.652 (0.527–0.776)	0.045	0.641	0.718	0.00932
CD63^+^-EVs × CA19-9	0.903 (0.823–0.984)	0.136	0.872	0.897	0.124
*Early stage (I–II)*					
CA19-9	0.814 (0.699–0.929)	11.7	0.769	0.87	
CD63^+^-EVs	0.858 (0.745–0.971)	0.011	0.0769	0.913	0.557
CD41^+^-EVs	0.685 (0.529–0.84)	0.079	0.744	0.738	0.197
CD61^+^-EVs	0.657 (0.51–0.804)	0.045	0.641	0.739	0.0992
CD63^+^-EVs × CA19-9	0.906 (0.802–1)	0.264	0.923	0.87	0.0330
*Late-stage (III–IV)*					
CA19-9	0.883 (0.773–0.993)	36	0.949	0.75	
CD63^+^-EVs	0.83 (0.689–0.972)	0.018	0.846	0.812	0.517
CD41^+^-EVs	0.668 (0.495–0.842)	0.081	0.744	0.625	0.00769
CD61^+^-EVs	0.644 (0.468–0.82)	0.048	0.667	0.688	0.00236
CD63^+^-EVs × CA19-9	0.899 (0.773–1)	0.136	0.872	0.875	0.809

ROC: Receiver operating characteristic, AUC: area under curve, CI: confidence interval

^a^*p*-value in DeLong's test

### Correlation analysis between marker-positive EVs and CA19-9

**Fig. 3:**
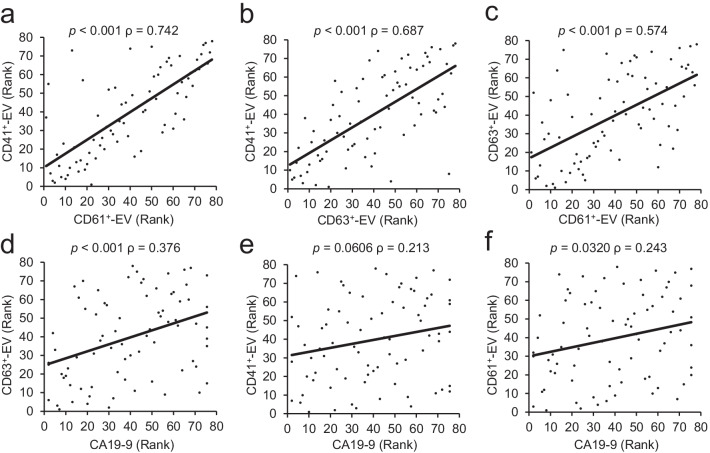
Correlation analysis between EVs and CA19-9. Spearman’s rank correlation coefficient tests between CD41^+^-EVs and CD61^+^-EVs (**a**), CD41^+^-EVs and CD63^+^-EVs (**b**), CD63^+^-EVs and CD61^+^-EVs (**c**), CD63^+^-EVs and CA19-9 (**d**), CD41^+^-EVs and CA19-9 (**e**), CD61^+^-EVs, and CA19-9 (**f**) were shown. ρ and *p*-values are indicated in the figure

To evaluate the strength of the relationship between serum EVs and CA19-9, we performed a non-parametric Spearman’s correlation analysis. As expected, CD41^+^-EVs and CD61^+^-EVs showed the highest correlation between them (ρ = 0.742), as both could be secreted from the same origin (Fig. 3a). CD41^+^-EVs and CD61^+^-EVs showed moderate correlation with CD63^+^-EVs (ρ = 0.687 and ρ = 0.574, respectively) (Fig. 3b, c). Conversely, all of the EVs (CD63^+^-EVs, CD41^+^-EVs, CD61^+^-EVs) showed only a low correlation with CA19-9 (ρ = 0.376, ρ = 0.213, ρ = 0.243, respectively) (Fig. 3d–f). A similar result was also observed in the parametric Pearson’s correlation analysis (see Additional file 1: Fig. S2). These results suggest that the serum EVs and CA19-9 are independent biomarkers of PDAC pathology.

### Diagnostic performance of the serum EVs in early- and late-stage PDAC

**Fig. 4:**
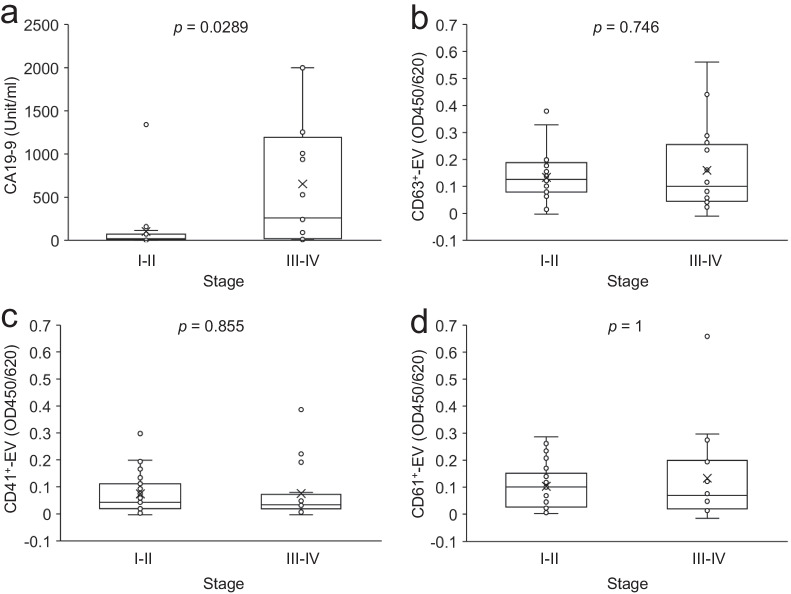
Comparison of the values of EVs and CA19-9 between early and late-stage PDAC. CA19-9 (**a**), CD63^+^-EVs (**b**), CD41^+^-EVs (**c**), and CD61^+^-EVs (**d**) were shown in early (I–II, n = 23) and late (III–IV, n = 16) clinical stage of patients with PDAC. p-values obtained by Wilcoxon–Mann–Whitney test are indicated in the figure

Early diagnosis of PDAC is crucial to improving the prognosis of PDAC. It has been reported that the diagnostic performance of CA19-9 in early-stage PDAC is lower than that in late-stage one [11]. Consistently, the serum level of CA19-9 in patients with early-stage (stage I–II) PDAC was significantly lower than that in late-stage (stage III–IV) patients with PDAC (Fig. 4a). ROC analysis of CA19-9 values also showed lower AUC for early-stage PDAC (AUC: 0.814) compared to late-stage PDAC (AUC: 0.883) to differentiate from HCs (Table 2). In contrast, the serum levels of CD63^+^-EVs, CD41^+^-EVs, and CD61^+^-EVs in early-stage PDAC were comparable to those in late-stage PDAC with *p* > 0.05 (*p* = 0.746, *p* = 0.855, *p* = 1, respectively, Fig. 4). The AUC values of CD63^+^-EVs, CD41^+^-EVs, and CD61^+^-EVs in early-stage PDAC (0.858, 0.685, 0.657, respectively) were also similar to those for late-stage PDAC (0.83, 0.668, 0.644, respectively) (Table 2). These results demonstrate that the serum levels of EVs are similar between early- and late-stage PDAC.

### ***Combinational use of CD63***^+^***-EVs and CA19-9 for early diagnosis of PDAC***

**Fig. 5:**
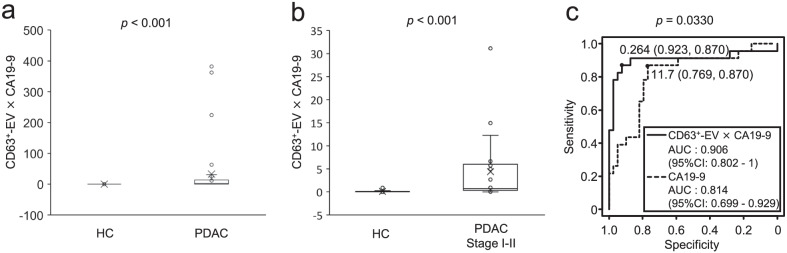
Combined use of CD63^+^-EVs and CA19-9 for detection of early-stage PDAC. The values of CD63^+^-EVs × CA19-9 were calculated by multiplying the OD values of CD63^+^-EVs and CA19-9 level. The values of CD63^+^-EVs × CA19-9 in HCs (n = 39) and PDACs (n = 39) (**a**) or early-stage PDACs (stage I–II, n = 23) (**b**) were shown. p-values obtained by Wilcoxon–Mann–Whitney Test are indicated in the figure. (**c**) Comparison of ROC curve of CA19-9 and CD63^+^-EVs × CA19-9 in early-stage sets consisting of 39 HCs and 23 early-stage PDAC. Solid and dotted line indicates CD63^+^-EVs × CA19-9 and CA19-9, respectively. p-values obtained by DeLong’s test are indicated in the figure

The low correlation coefficient of serum levels between CA19-9 and CD63^+^-EVs suggested that the combined use of these markers may compensate for the false-negative with each other and improve the diagnostic performance. Therefore, we examined the diagnostic performance of cobitional use of CA19-9 and CD63^+^-EVs. In order to weight the value of CA19-9 and CD63^+^-EVs equally, we used the value obtained by multiplying these two values as CA19-9 × CD63^+^-EVs. The value of CA19-9 × CD63^+^-EVs was significantly higher in patients with PDAC than in HCs (Fig. 5a). Remarkably, CA19-9 × CD63^+^-EVs showed a significantly higher AUC for early-stage PDAC (stage I–II, AUC: 0.906) than CA19-9 (AUC: 0.814, *p* = 0.033) (Fig. 5b, c). In contrast, the AUC values of CA19-9 × CD63^+^-EVs for all-stage (0.903) and late-stage PDAC (0.899) were comparable to those of CA19-9 (all stage: AUC = 0.842, *p* = 0.124; late-stage: AUC = 0.883, *p* = 0.809) (Table 2). These results indicate that the combined use of CD63^+^-EVs and CA19-9 improves the diagnostic performance for early-stage PDAC (I–II).

### Influence of surgical resection on the serum levels of EVs

**Fig. 6:**
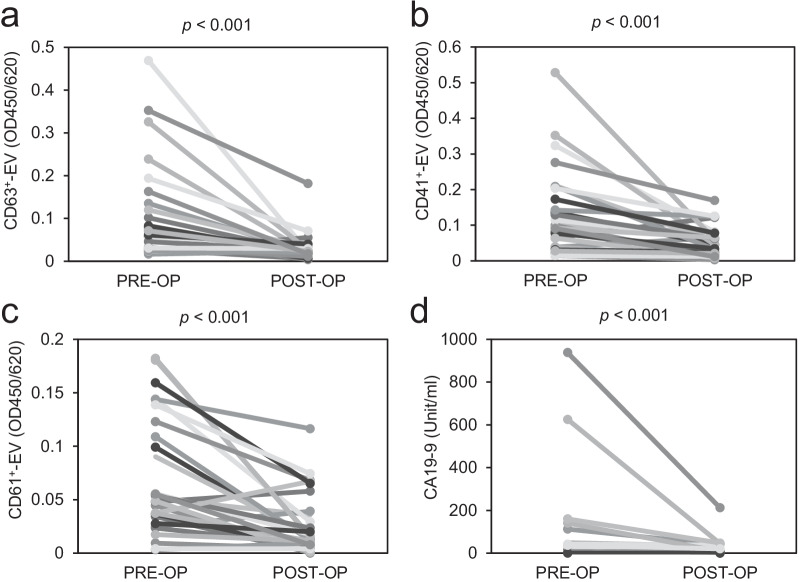
Effect of surgical resection on the serum levels of EVs and CA19-9. The values of CD63^+^-EVs (**a**), CD41^+^-EVs (**b**), CD61^+^-EVs (**c**) and CA19-9 (**d**) before (PRE-OP) and after operation for 30–90 days (POST-OP) of patients with PDAC (n = 28 for (**a**), (**b**), (**c**), n = 19 for (**d**)). *p*-values obtained by Wilcoxon signed rank test are indicated in the figure

Finally, we investigated the effect of surgical resection on the serum levels of EVs. The serum levels of CA19-9, CD63^+^-EVs, CD41^+^-EVs and CD61^+^-EVs were significantly decreased after surgical resection (Fig. 6a–d).

Of 28 patients with PDAC, 14 patients had recurrence and 14 patients had non-recurrence after surgical recession. We examined whether preoperative EV levels predict the recurrence of PDAC. Recurrence group showed a trend to higher CD41^+^-EV and and CD61^+^-EV levels in comparison with non-recurrence group, but not statistically significant (Additional file 1: Fig. S3, *p* = 0.0620 and 0.0620, respectively).

We further analyzed the change in the values of EVs and CA19-9 before and after operation in each patient (see Additional file 1: Fig. S4). The serum levels of EVs and CA19-9 fluctuated in the same trend before and after surgical resection in most patients except for patient’s No. 7, 8, and 16. This result suggests that the serum levels of EVs reflect tumor burden similar to CA19-9. The inconsistency in patients No. 7 and 8 is likely due to the low serum level of CA19-9 in the preoperative stage (4.8 and 3.5 unit/ml, respectively; threshold: 11.7), while the serum level of CD63^+^-EVs was high at this stage (0.0596 and 0.0216, respectively; threshold: 0.016). These results demonstrate that the serum levels of platelets (CD41 and CD61) and universal marker (CD63)-positive EVs reflect the tumor burden before and after surgical resection.

## Discussion

There are many reports on the functions and medical applications of PDAC-derived EVs [7]. Here we have demonstrated that both platelet markers (CD41, CD61)-positive EVs and CD63-positive EVs are increased in sera of patients with PDAC. The serum levels of CD63^+^-EVs showed diagnostic performance comparable to CA19-9, while there is a low correlation between them. The combined use of CA19-9 and CD63^+^-EVs showed a higher AUC for early-stage PDAC compared to CA19-9. Interestingly, the serum EVs levels reflected the tumor burden, indicating the association of this EV production with tumors.

Among the serum EVs measured in this study, CD63^+^-EVs showed the highest performance in discriminating patients with PDAC and HCs. CD63, a member of the tetraspanin family, is highly enriched in the exosome subpopulation, thus, generally recognized as an exosome marker [12]. The serum CD63^+^-EV levels reflected the tumor burden since they decreased after surgical resection and fluctuated together with CD19-9 levels. Recently, Hoshino et al. investigated the proteomic profile of EVs and extracellular particles in 426 human samples from tissue explants, plasma, and other body fluids [13]. CD63^+^-EVs were rarely detected in either human or mouse origin through biofluids (plasma, serum, lymphatic fluid). This observation is consistent with our results that the serum levels of CD63^+^-EVs in HCs are low [9]. Consistently, CD63^+^-EVs were previously reported to be increased in colorectal cancer patients compared to HCs [14]. We previously reported that CD63^+^-EVs are increased in sera of AD patients. Therefore CD63^+^-EVs was shown to respond to neurodenegerative disorders as well as cancers.

One of the origins of CD63^+^-EVs is believed to be platelets. In sera of both PDAC and AD patients, CD63^+^-EVs showed a positive correlation with CD41^+^-EVs and CD61^+^-EVs derived from platelets. The serum levels of CD41^+^-EVs and CD61^+^-EVs were significantly increased in sera of patients with PDAC and decreased after surgical resection. It was notable that CD41^+^-EVs and CD61^+^-EVs fluctuated with the same trend as CD19-9 before and after surgical resection. Therefore, platelet-derived EVs might be directly or indirectly associated with PDAC. Growing evidence demonstrates that activated platelet and platelet-derived EVs have a critical role in cancer metastasis [15]. “Tumor-educated platelets” are involved in the progression and spread of several tumors and can be used as potential biomarkers for cancer detection [16, 17]. An in vitro study demonstrated that cancer cells, including PDAC cell lines, can activate platelets [18]. Therefore, EVs released from tumor-educated platelets might be related to cancer progression and metastasis. Consistently, the formation of the aggregates of platelet, tumor, and platelet-derived EVs has been suggested to assist the microvascular arrest of cancer cells during the metastasis process [15, 19].

CD63^+^-EVs demonstrated high diagnostic performance in discriminating PDAC from HCs. Thus, there are potential benefits for the clinical use of CD63^+^-EVs. First, combined with CA19-9, CD63^+^-EVs can increase the performance to discriminate early-stage patients with PDAC, as shown in Fig. 5c. Second, CD63^+^-EVs can be used to evaluate the therapeutic effects of surgical and drug treatments. As described above; however, CD63^+^-EVs are not specific to PDAC but increase in other diseases [9]. Therefore, we hypothesize that CD63^+^-EVs might increase in sera of patients with various diseases. In our preliminary experiments it was observed that serum levels of CD63^+^-EVs increased in sera of patients with various cancers, inflammatory diseases, and neurodegenerative/mental disorders. Although the mechanism by which CD63^+^-EVs increases in blood is unclear, it might be linked to disease-related inflammation. In this sense, CD63^+^-EVs could be used as an inflammatory-associated disease biomarker. To apply CD63^+^-EVs for diagnosis, in future studies we need to understand the mechanism by which CD63^+^-EVs increases in sera of patients and their functions.

## Conclusions

In this study, we showed that the serum levels of universal CD63^+^-EVs and platelet-derived EVs (CD41^+^-EVs, CD61^+^-EVs) are increased in patients with PDAC than HCs. The serum levels of CD63^+^-, CD41^+^, and CD61^+^ EVs reflected the tumor burden in patients with PDAC. Since CD63^+^-EVs showed a high AUC to discriminate patients with PDAC from HCs; they might be useful as potential biomarkers for PDAC.

## Supplementary Information

**Additional file 1.** Fig. S1–S4.

**Additional file 2: Table S1.** Summary of Participant's characteristics of demographic data.

**Additional file 3: Table S2.** Summary of Power calculation.

## Data Availability

The datasets used for the current study are available from the corresponding author on reasonable request.

## Abbreviations

PDAC: Pancreatic ductal adenocarcinoma
EV: Extracellular vesicle
AUC: Area under curve
AD: Alzheimer’s disease
HC: Healthy control
ELISA: Enzyme-linked immunosorbent assay
HRP: Horseradish peroxidase
OD: Optical density
ROC: Receiving operator curve
PRE-OP: Preoperation
POST-OP: Postoperation
