# Supplementary material for: CD63-positive extracellular vesicles are potential diagnostic biomarkers of pancreatic ductal adenocarcinoma
Source: BMC Gastroenterol. 2022 Mar 28;22:153. doi: 10.1186/s12876-022-02228-7 (PMC8962497; doi:10.1186/s12876-022-02228-7)
Supplement: Supplementary file 1 — Additional file 1. Fig. S1–S4. [file 12876_2022_2228_MOESM1_ESM.pdf]

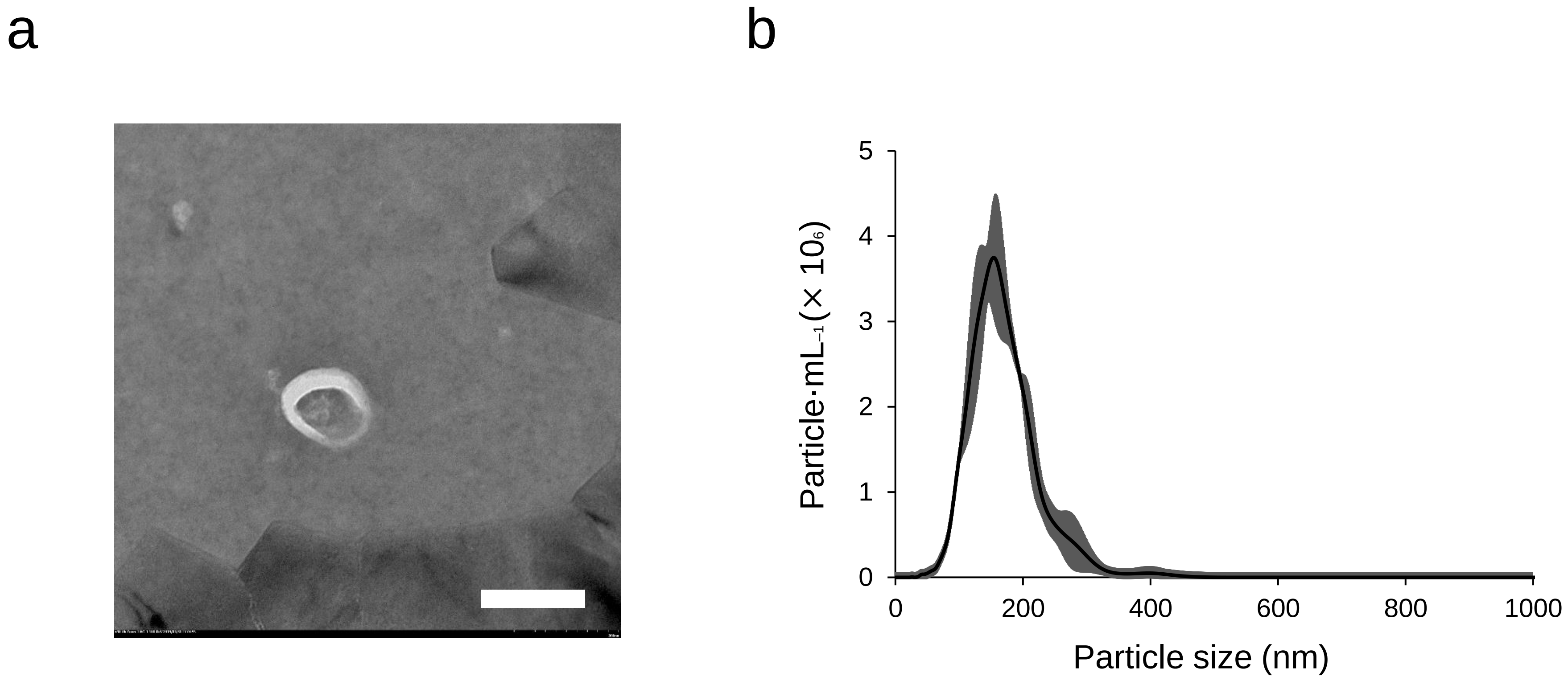


**Fig. S1** Characterization of serum-derived EVs from PDAC patients. (a) Representative TEM images of EVs isolated from sera of PDAC. Scalebar: 200nm (b) Size distribution of PDAC-derived EVs analyzed by NTA (n = 3). Gray area indicates SD.

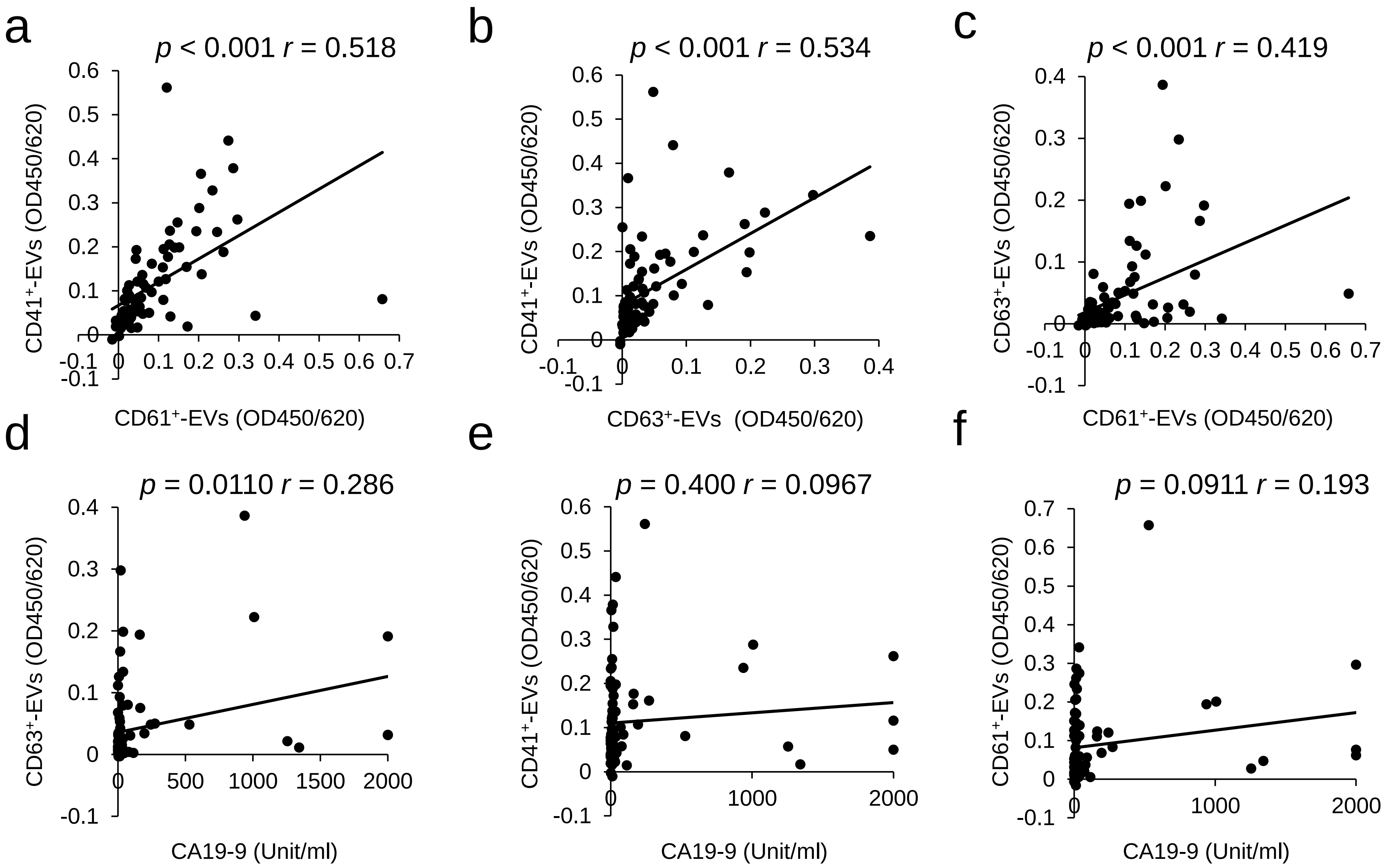


**Fig. S2** Parametric pearson's correlation analysis between EVs and CA19-9. Pearson's correlation coefficient tests between $CD41^+$-EVs and $CD61^+$-EVs (a), $CD41^+$-EVs and $CD63^+$-EVs (b), $CD63^+$-EVs and $CD61^+$-EVs (c), $CD63^+$-EVs and CA19-9 (d), $CD41^+$-EVs and CA19-9 (e), $CD61^+$-EVs and CA19-9 (f) were shown. r and p-values were indicated in the figure.

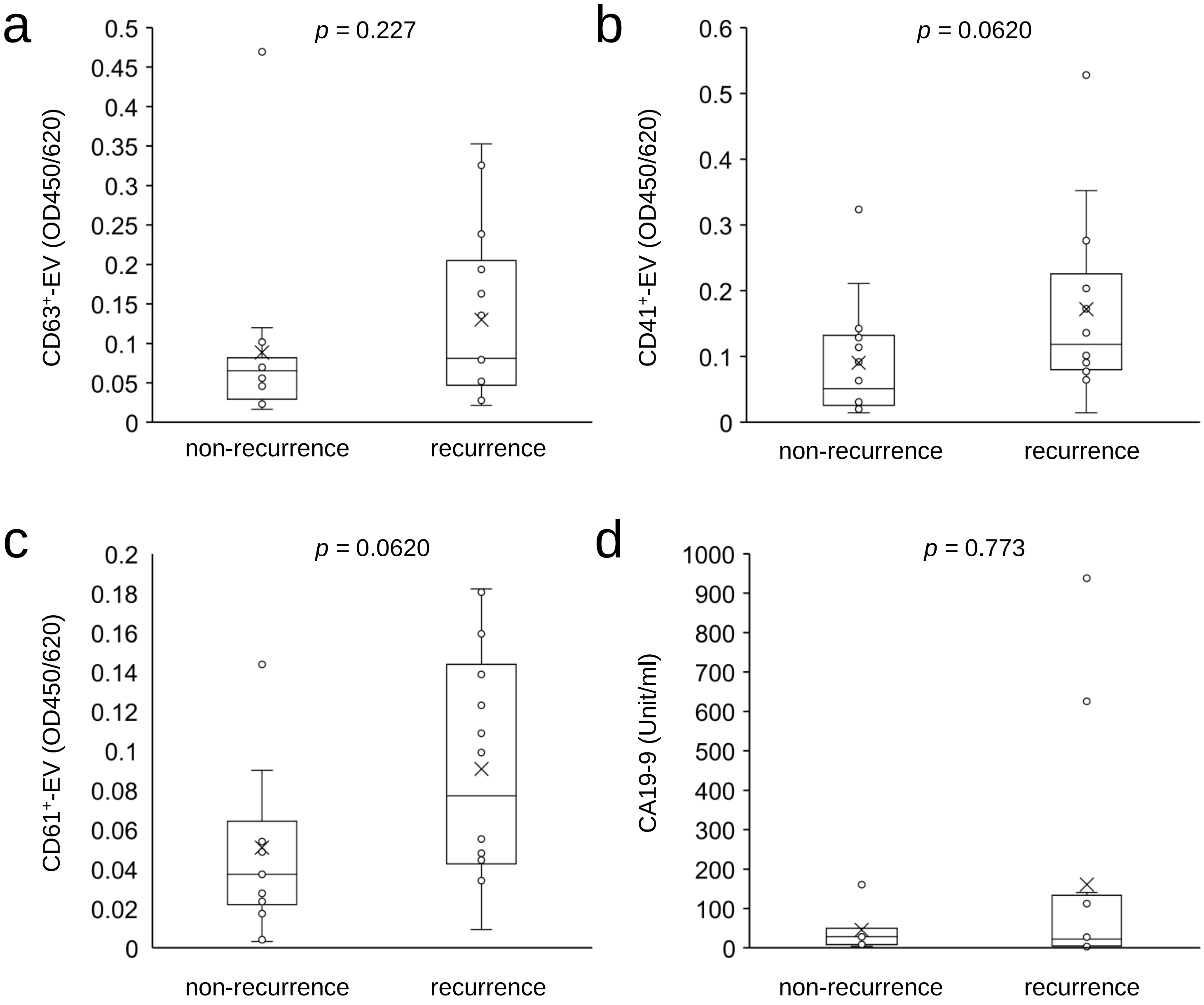


**Fig. S3** The pre-operative EVs and CA19-9 levels in non-recurrence and recurrence patients. CD63+-EVs (a), CD41+-EVs (b), and CD61+-EVs (c) and CA19-9 (d) were shown in non-recurrence (a-c: n = 14, d: n=7) and recurrence (a-c: n = 14, d: n=12) patients with PDAC. p-values obtained by Wilcoxon–Mann–Whitney Test are indicated in the figure.

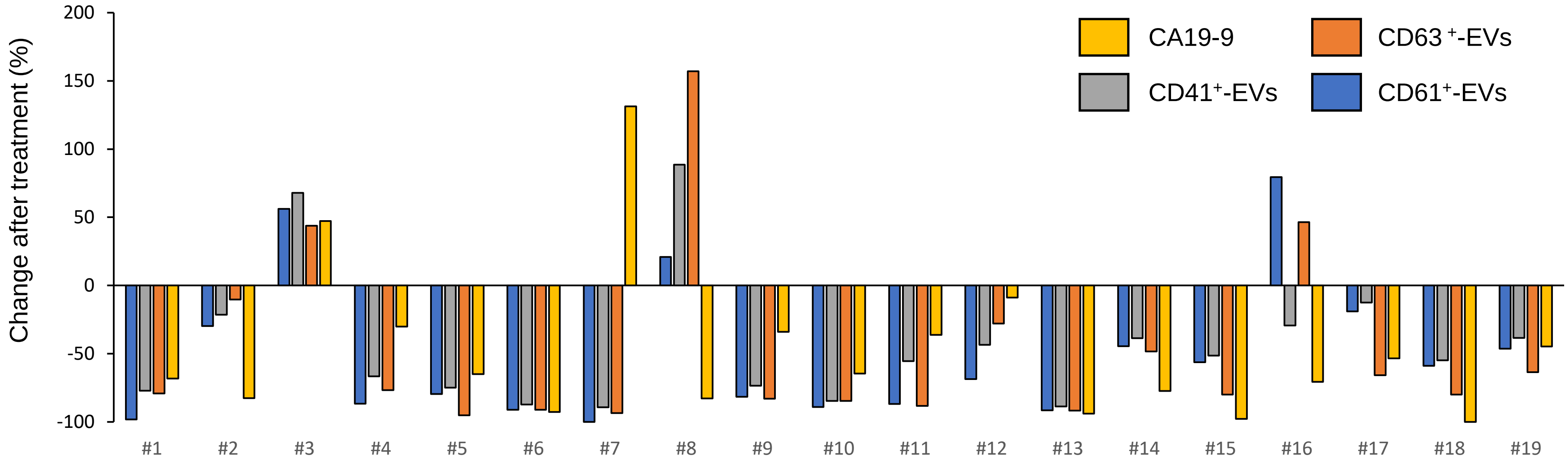


**Fig. S4** The serum levels of EVs and CA19-9 before and after surgical resection. Changes in the serum levels of EVs and CA19-9 compared to pre-surgical resection in each patient were calculated and were shown as percentage.
