# Supplementary material for: CD63-positive extracellular vesicles are potential diagnostic biomarkers of pancreatic ductal adenocarcinoma
Source: BMC Gastroenterol. 2022 Mar 28;22:153. doi: 10.1186/s12876-022-02228-7 (PMC8962497; doi:10.1186/s12876-022-02228-7)
Supplement: Supplementary file 3 — Additional file 3: Table S2. Summary of Power calculation. [file 12876_2022_2228_MOESM3_ESM.docx]

**Table S2. Summary of Power calculation**

|  | | Difference in average | Standard division | Power^a^ |
| --- | --- | --- | --- | --- |
| Cohort 1  (N=39) | CA19-9 | 316.96411 | 306.781515 | 0.995 |
|  | CD63^+^-EVs | 0.06192222 | 0.055190365 | 0.999 |
|  | CD41^+^-EVs | 0.06054445 | 0.10188981 | 0.747 |
|  | CD61^+^-EVs | 0.05251026 | 0.099967465 | 0.64 |
| Cohort 2  (N=28 in EVs, N=19 in CA19-9) | CA19-9 | 90.17368 | 145.7734 | 0.723 |
|  | CD63^+^-EVs | 0.07806191 | 0.07238764 | 1 |
|  | CD41^+^-EVs | 0.0789619 | 0.081676 | 0.998 |
|  | CD61^+^-EVs | 0.04404881 | 0.041997075 | 1 |

^a^ *α* is set at 0.05
